# Galectins: Role and Therapeutics in Diabetes and Diabetic Foot Ulcers

**DOI:** 10.3390/biom16020232

**Published:** 2026-02-03

**Authors:** Alhasan Alobaidi, Rawan Al Judeid, Vikrant Rai

**Affiliations:** 1 Department of Translational Research, College of Osteopathic Medicine of the Pacific, Western University of Health Sciences, Pomona, CA 91766, USA; alhasan.alobaidi@westernu.edu; 2 Department of Biological Sciences, Citrus College, Glendora, CA 91741, USA

**Keywords:** diabetes, diabetic complications, diabetic foot ulcers, galectins, therapeutic targets

## Abstract

Diabetes is a chronic inflammatory disease due to decreased insulin release or insulin resistance. Diabetes complications stem from high blood sugar damaging blood vessels and nerves, leading to issues like heart disease, stroke, kidney failure, nerve damage, vision loss, foot ulcers, gum disease, skin infections, and digestive/bladder issues. Galectins, especially galectin-3, are emerging as key players in diabetes complications, promoting fibrosis, inflammation, and vascular damage. This suggests that galectins may be potential therapeutic targets in diabetes and its complications, and a need to understand their role and therapeutic potential. The objective of this review is to synthesize current evidence on galectin biology in diabetes mellitus (mainly on type II diabetes) and DFUs, delineate their mechanistic roles in metabolic dysfunction and wound healing, summarize findings from human and preclinical studies, and evaluate emerging diagnostic and therapeutic strategies. Finally, this review highlights key gaps that must be addressed to advance clinical translation. The literature search suggests that galectins play a critical role in the pathogenesis of diabetes and related complications and may be potential therapeutic targets.

## 1. Introduction

Diabetes mellitus (DM) is a chronic metabolic disorder characterized by persistent hyperglycemia resulting from impaired insulin secretion, defective insulin action, or both [1]. This prevalent endocrine disease contributes substantially to global morbidity and mortality. Among its most debilitating complications are diabetic foot ulcers (DFUs), defined as ulceration, infection, or destruction of foot tissues associated with peripheral neuropathy and/or peripheral artery disease (PAD) [2]. Globally, individuals with diabetes face a lifetime risk of developing a DFU of approximately 19–34%, with an estimated 18.6 million new cases annually [3,4]. DFUs are a major cause of morbidity, with approximately 20% of cases progressing to lower-extremity amputation and representing a leading cause of non-traumatic limb loss in people with diabetes. They are also associated with high mortality, with five-year survival rates of approximately 50.9%, significantly lower than in individuals with diabetes without foot ulcers [3,5].

The pathogenesis of DFUs involves interconnected metabolic, inflammatory, vascular, and microbiological abnormalities with chronic hyperglycemia, immune dysregulation, impaired angiogenesis, and persistent infection. Chronic hyperglycemia promotes oxidative stress, sustained inflammation, endothelial dysfunction, and impaired immune responses, collectively contributing to defective wound healing [5,6,7,8]. In addition, DFU wounds frequently harbor polymicrobial biofilms encased within a protective polymeric matrix, which confers resistance to immune clearance and antimicrobial therapy and perpetuates chronic non-healing ulcers. Failure to resolve these wounds markedly increases the risk of infection, gangrene, and limb loss [2,8,9].

Galectins are a highly conserved family of β-galactoside-binding proteins that translate glycan-encoded information into critical cellular processes, including apoptosis, cell proliferation, migration, and tissue homeostasis [10,11,12,13]. These multifunctional proteins are central regulators of chronic inflammatory and metabolic disorders, particularly obesity and type 2 diabetes mellitus (T2DM), where metaflammation precedes disease onset [14,15]. Several family members contribute to metabolic regulation: Galectin-3 (Gal-3), frequently elevated in obesity and T2DM, has been implicated in insulin resistance, whereas Galectin-1 (Gal-1) and Galectin-9 (Gal-9) modulate immune responses, and Galectin-12 (Gal-12) regulates lipid turnover and adipocyte function [14,16,17].

Gal-3 exhibits a striking duality: systemically elevated levels correlate with insulin resistance and fibrosis in complications like nephropathy and cardiomyopathy [18,19]. However, locally in DFUs, its crucial pro-angiogenic function (mediated by integrin α5β1) is aberrantly blocked by accumulating Advanced Glycation End-products (AGEs) [20,21]. Conversely, Gal-1 accelerates pathological wound healing by initiating myofibroblast activation and promoting pro-resolving macrophage polarization [22]. Furthermore, crucial epithelial repair is hampered by the impaired expression of Galectin-7 (Gal-7) in diabetic keratinocytes, a defect linked directly to hyperglycemia-induced modifications [23]. These complex, context-dependent roles solidify Gal-3 as a critical prognostic biomarker for complications like diabetic kidney disease (DKD) and DFU [24,25]. Future therapeutic success hinges on resolving the spatial paradox through highly selective strategies, such as the local delivery of recombinant Gal-3 via hydrogels to enhance localized regeneration while mitigating systemic side effects [20,22]. Integrating advanced techniques, including single-cell RNA sequencing (scRNA-seq) and detailed longitudinal studies, is essential to validate galectin-targeted interventions and facilitate personalized management within a precision medicine framework [6,26,27].

The convergence of metabolic dysfunction, chronic inflammation, and impaired wound repair positions galectins as key integrators within the diabetic microenvironment. Through their effects on immune cell phenotypes, fibroblast activation, angiogenesis, and cell migration, galectins influence processes critical to wound healing [12,17,22]. Persistent hyperglycemia further promotes the accumulation of advanced glycation and lipoxidation end products (AGEs/ALEs), which modify galectin-dependent signaling pathways and contribute to delayed wound repair in DFUs [27,28]. Despite these connections, the specific and coordinated contributions of major galectins to diabetic wound repair remain under-synthesized. While individual studies have examined galectins in metabolic tissues, immune cells, or isolated wound models, an integrated framework linking metabolic dysregulation, chronic inflammation, and DFU pathobiology is lacking [6,26,27]. Altered galectin expression, therefore, holds promise as both a prognostic biomarker and therapeutic target in metabolic disease and its complications [13,18,29,30]. Galectins emerge as pivotal, multifaceted mediators regulating these cellular and metabolic abnormalities at the core of the diabetic microenvironment [31]. This comprehensive review synthesizes current evidence regarding major galectins to elucidate their intricate mechanistic roles, evaluate their potential as biomarkers, and assess emerging therapeutic strategies in DM and DFU [14,31].

## 2. The Galectin Family: Structure, Biology, and Distribution

### 2.1. Galectin Classification and Structural Biology

**Figure 1:**
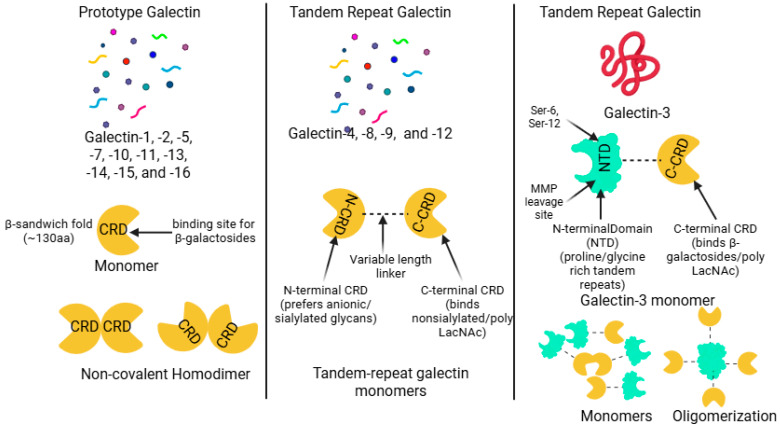
Structural classification of the galectin family. Galectins are grouped into prototype, tandem-repeat, and chimera types based on carbohydrate recognition domain (CRD) number and organization. Prototype galectins form non-covalent homodimers, tandem-repeat galectins contain two distinct CRDs connected by a linker, and Galectin-3 uniquely possesses a non-lectin N-terminal domain that mediates oligomerization. Created in BioRender. Rai, V. (2026) https://BioRender.com/cuz1cwb (accessed on 19 December 2025).

Galectins are a family of soluble lectins expressed across the animal kingdom, defined by their conserved carbohydrate recognition domain (CRD) and specific affinity for β-galactoside-containing glycans [12,29,32,33]. This highly conserved CRD typically comprises around 130 amino acids and folds into an intricate β-sandwich structure formed by two antiparallel β-sheets [34,35]. Structurally, galectins are broadly categorized into three types: prototype (single CRD, forms homodimers), tandem-repeat (two distinct CRDs linked by a peptide), and chimera type (single CRD linked to a non-lectin N-terminal domain that mediates oligomerization) [13,30,36] (Figure 1).

Prototype galectins contain one CRD per subunit and characteristically form non-covalent homodimers [30,32]. Members of this class generally include Galectin-1, -2, -5, -7, -10, -11, -13, -14, -15, and -16 [26,30,37]. Tandem-repeat galectins possess two homologous but distinct CRDs connected by a variable linker segment within a single polypeptide chain [38,39,40]. This structural group includes Galectin-4, -6, -8, -9, and -12 [16,21,26]. Galectin-3 is the sole member of the chimera-type class [21,26,30,40]. It is structurally unique, being composed of a C-terminal CRD connected to a flexible N-terminal non-lectin domain characterized by extensive proline- and glycine-containing repeats [26,30,40,41,42]. This N-terminal domain is crucial as it facilitates oligomerization, often resulting in the formation of multivalent pentamers when interacting with glycoconjugates [26,40,41,43] (Figure 1).

CRD is defined by a β-sandwich fold typically comprising approximately 130 amino acids. This structure consists of two antiparallel β-sheets, specifically categorized as the sugar-binding S-face and the opposing F-face, where the glycan interaction primarily takes place [30,34,44]. Glycan binding occurs within a shallow groove located along the S-face, supported by an extensive network of highly specific hydrogen bonding and crucial hydrophobic CH–π stacking interactions, notably involving a conserved Tryptophan residue. This binding groove is functionally compartmentalized into typically five distinct subsites (A–E) [35,45,46,47]. The most highly conserved portion is Subsite C, which serves to anchor the core β-galactose moiety, while the variability found in peripheral subsites (A, B, and E) introduces the functional distinctions that dictate precise ligand specificity among different galectins [35,45,47]. The minimal carbohydrate recognized is the β-galactoside unit, with N-acetyllactosamine (LacNAc) recognized as the fundamental core recognition disaccharide. Affinity significantly increases in proportion to the presence of repeating LacNAc motifs or β1-6-branched N-glycans. Furthermore, this interaction is tightly regulated by post-translational modifications, as terminal α2,6-sialylation typically inhibits binding for most family members [10,13,37,40,48]. However, Gal-3 possesses a unique ability to retain affinity by recognizing internal LacNAc structures, thus allowing it to bypass this terminal sialylation blockade [48,49,50] (Figure 1).

### 2.2. Expression Patterns of Major Galectins in Human Tissues

#### 2.2.1. Prototype Galectins (Gal-1, -2, -7, -10, -13, -14, -16)

Gal-1 is widely expressed in epithelial, adipose, vascular, and immune tissues and functions as a potent immunosuppressive mediator in inflammatory states [17,51,52]. Gal-1 induces apoptosis of activated T cells, particularly Th1 and Th17 subsets, through CD45-dependent signaling, thereby promoting immune tolerance and a Th2-skewed cytokine profile [31]. Circulating Gal-1 levels correlate positively with obesity, hyperinsulinemia, and insulin resistance, and experimental models show that Gal-1 exacerbates diet-induced obesity by enhancing peroxisome proliferator-activated receptor gamma) (PPARγ) transcriptional activity [53,54,55]. Gal-1 also exhibits pro-fibrotic activity by activating myofibroblasts via neuropilin-1/Smad3 signaling and is highly expressed in diabetic renal tubular cells, where it contributes to fibrosis and atherosclerotic smooth muscle cell migration [44,56,57]. In diabetic retinopathy, Gal-1 is markedly upregulated and acts as a pro-angiogenic factor, correlating positively with vascular endothelial growth factor (VEGF) levels in proliferative disease [26,58].

Galectin-2 (Gal-2) is primarily expressed in the gastrointestinal epithelium, cardiovascular tissue, and placenta, where it supports mucosal barrier integrity, potentially through mucin glycoprotein crosslinking [59,60]. Immunologically, Gal-2 can induce T-cell apoptosis and promote a Th2-skewed response but may also drive proinflammatory M1 macrophage polarization in context-dependent settings [31,60]. Genetic variants influencing Gal-2 expression are associated with increased cardiovascular risk and elevated fasting glucose and insulin levels [60]. In gestational diabetes mellitus, placental Gal-2 expression is consistently upregulated and is associated with altered glucose–insulin regulation and impaired fetal vascular development [61,62].

Galectin-7 (Gal-7) is a prototype galectin predominantly expressed in stratified epithelia, including the epidermis and cornea, where it promotes re-epithelialization by enhancing keratinocyte proliferation and migration during tissue repair [23,63,64]. In diabetic conditions, hyperglycemia suppresses Gal-7 expression, impairing wound healing, while Gal-7 also functions as an immune regulator capable of inducing T-cell apoptosis [23,31].

Galectin-10 (Gal-10) is highly expressed in eosinophils and forms Charcot–Leyden crystals, which act as alarmins when phagocytosed by macrophages, triggering NOD-, LRR-, and pyrin domain-containing protein 3 (NLRP3) inflammasome activation and IL-1β release [65].

Gal-13, -14, and -16 are predominantly expressed at the maternal-fetal interface in tissues like the placenta. These placental galectins promote immune tolerance necessary for a healthy pregnancy by inducing the apoptosis of activated T-cells [31,37,66].

#### 2.2.2. Chimeric Galectin (Gal-3)

Gal-3 contains a CRD linked to a non-lectin N-terminal domain [30]. It is expressed in epithelial cells, fibroblasts, and immune cells. Gal-3 is a central pro-inflammatory and pro-fibrotic mediator in cardiac, hepatic, renal, and pulmonary fibrosis. It enhances monocyte recruitment, angiogenesis, and atherosclerotic plaque development. Gal-3 correlates with insulin resistance and T2DM, possibly through direct insulin receptor interactions [30,41,59]. It also binds AGEs, potentially limiting certain diabetic complications [31,67].

#### 2.2.3. Tandem-Repeat Galectins (Gal-4, -8, -9, -12)

Gal-4 is enriched in gastrointestinal epithelium [68]. It stabilizes lipid rafts, regulates apical trafficking, and promotes intestinal wound healing. It also modulates immune responses via CD14 on monocytes. Elevated serum Gal-4 is linked to coronary heart disease and ischemic stroke. It may influence diabetes risk by enhancing dipeptidyl peptidase 4 ((DPP-4) activity and reducing glucagon-like peptide (GLP)-1 action [59,68,69].

Gal-8 is expressed in immune tissues, adipose tissue, and the GI tract [31,35]. Inside cells, it senses membrane damage and initiates antibacterial autophagy. Extracellularly, it activates nuclear factor kappa beta (NF-κB)–mediated cytokine and chemokine secretion [35,70]. Gal-8 expression also correlates with cartilage degeneration in osteoarthritis [71].

Gal-9 is widely expressed in immune tissues. Through T cell immunoglobulin and mucin domain-containing protein 3 (TIM-3) interactions, it induces apoptosis in Th1 and Th17 cells, regulating immune tolerance. Circulating Gal-9 reflects disease severity in several inflammatory disorders. It is also present in adipose tissue, where it modulates macrophage–T-cell communication [31,40,72].

Gal-12 is expressed mainly in adipocytes and sebocytes [73]. It regulates adipocyte differentiation and lipid turnover. Gal-12 deficiency enhances lipolysis and improves glucose tolerance in obese mice. In sebocytes, it regulates lipogenesis through PPARγ-dependent pathways. Loss of Gal-12 also reduces atherosclerosis by promoting M2 macrophage polarization and limiting foam-cell formation [31,73,74,75,76].

## 3. Galectins: General Effect Pathways and Diabetes

Galectins recognize β-galactoside-containing glycans via their CRDs and interpret the structural information encoded in cell-surface and extracellular glycoconjugates [10,12,13,30]. They are localized to the cytoplasm, nucleus, extracellular matrix, and intercellular spaces. Extracellular galectins bind glycoproteins in bivalent or multivalent configurations, forming receptor arrays known as galectin lattices [12,34,77]. Through these mechanisms, galectins influence cell adhesion, migration, differentiation, and tissue remodeling. Intracellular galectins participate in protein–protein interactions independent of carbohydrate, serving as scaffolds for key regulators like K-RAS and Bcl-2 family members to promote cell survival and proliferation [37,77,78]. They function as innate danger signal sensors that recognize exposed glycans on damaged endolysosomes and initiate cellular repair or autophagy [12,79]. This broad regulatory spectrum explains their pervasive involvement in pathologies such as cancer, chronic inflammation, fibrosis, and impaired diabetic wound healing [80,81]. Table 1 summarizes the location and the role of various galectins in diabetes and its complications.

### 3.1. Galectins in Glucose Metabolism

While DM encompasses distinct subclasses with unique pathogeneses, galectins play divergent biological roles depending on the specific etiology of the disease. In T1DM, which is driven by autoimmunity, Gal-3 acts as a pro-inflammatory mediator facilitating pancreatic β-cell destruction, whereas Gal-1 and Galectin-9 (Gal-9) exhibit protective immunomodulatory effects [14,88]. In contrast, in T2DM, elevated Gal-3 contributes directly to systemic insulin resistance, while Gal-12 regulates adipocyte function and inflammation [14,89]. Additionally, specific alterations such as placental Gal-2 upregulation and elevated serum Gal-3 characterize Gestational Diabetes Mellitus (GDM) [62]. However, the local pathophysiology of DFUs discussed in this review is a convergent pathway applicable to all diabetes types. The accumulation of AGEs, which blocks the pro-angiogenic function of Gal-3 in wounds, is a direct consequence of the chronic hyperglycemia shared by T1DM, T2DM, and uncontrolled GDM [20,21,88]. Multiple members of the galectin family directly regulate glucose homeostasis and energy metabolism, with Gal-1, -3, -4, and -12 most consistently implicated in obesity and T2DM [31,59,65,74] (Table 2).

Circulating Gal-1 levels are also frequently elevated in obesity and correlate positively with BMI, fasting insulin, and HOMA-IR. However, longitudinal data suggest that Gal-1 may exhibit stage-dependent or compensatory behavior during disease progression, indicating context-specific metabolic effects rather than a uniformly pathogenic role [54,55]. Gal-1 exhibits a nuanced metabolic and generally anti-inflammatory profile. Experimental models suggest that Gal-1 supports glucose homeostasis primarily by enhancing glucose-stimulated insulin secretion (GSIS) from pancreatic β cells. Specifically, Gal-1 deficiency resulted in impaired glucose tolerance in female mice, while the administration of recombinant Gal-1 effectively augmented insulin release, pointing to a protective role in β-cell function, even though circulating Gal-1 is associated with obesity [17,55].

Recent comprehensive reviews have further solidified the role of Gal-1 as a central mediator linking obesity, T2DM, and complications. Fryk et al. [95] highlighted that circulating and adipose tissue Gal-1 levels are consistently elevated in obesity, correlating positively with BMI, insulin resistance, and pro-inflammatory markers such as TNF- and IL-6. While this elevation may initially serve as a compensatory mechanism to regulate adipogenesis and lipogenesis, the chronic inflammatory environment of T2DM eventually overwhelms these protective effects. Thiemann and Baum [96] further emphasized that galectins are critical translators of glycan-encoded information that regulate immune cell homeostasis; this immunometabolic crosstalk is essential for maintaining tissue integrity. In the specific pathophysiology of DFUs, Gal-1′s role extends beyond metabolism; it is implicated in the regeneration of peripheral nerves and cutaneous wound healing, suggesting that its dysregulation in obesity and T2DM directly contributes to the neuropathic and non-healing phenotype of diabetic foot ulcers [95].

Clinical studies show that circulating Gal-3 levels are frequently elevated in obesity and correlate with insulin resistance indices, including body mass index (BMI) and Homeostatic Model Assessment for Insulin Resistance (HOMA-IR) [82,97]. Gal-3 is a key mediator of systemic insulin resistance. Extracellular Gal-3 directly binds the insulin receptor (InsR), impairing receptor autophosphorylation and downstream signaling. This disruption results in reduced glucose transporter type 4 (GLUT4) translocation and diminished insulin-stimulated glucose uptake in adipocytes, hepatocytes, and myocytes [92,93]. In parallel, intracellular Gal-3 modulates metabolic signaling by sensing lipopolysaccharide (LPS) and activating mechanistic target of rapamycin complex 1 (mTORC1)-dependent pathways that upregulate glycolytic enzymes, including glucose transporter type 1 (GLUT1), hexokinase 2 (HK2), and pyruvate kinase M2 (PKM2) [90,91,92]. This highlights a dual extracellular–intracellular role in metabolic reprogramming [91] (Table 2).

Gal-4 mainly influences glucose regulation indirectly through its actions involving gastrointestinal mechanisms. This galectin is believed to stabilize lipid rafts necessary for the apical trafficking of DPP-4, an enzyme that rapidly inactivates incretin hormones. Consequently, elevated Gal-4 levels are proposed to increase diabetes risk by enhancing DPP-4 activity, thereby diminishing incretin-mediated insulin secretion [68,83,84] (Table 2).

Gal-12 functions primarily as a negative regulator of lipid metabolism. This galectin is localized to lipid droplets within adipocytes and acts to suppress lipolysis, thereby regulating energy expenditure. Its genetic ablation leads to reduced adiposity and improved insulin sensitivity in obese animal models, establishing Gal-12 as a critical mediator between adipocyte lipid handling and systemic glucose control [73] (Table 2).

Collectively, these data position various galectins as central integrators of β-cell function, insulin signaling, adipocyte lipid metabolism, and enteroendocrine regulation. Their intricate, context-dependent actions emphasize the need to carefully distinguish between systemic and tissue-specific effects when evaluating galectins as metabolic biomarkers or therapeutic targets for diabetes [14,30,55,68].

### 3.2. Galectins in Inflammation and Immune Modulation

Galectins are expressed by various immune cells and have key roles in immune cell homeostasis. Galectin activity has context-dependent outcomes that are both pro-inflammatory and anti-inflammatory, influenced by cell type, location, and disease state [12,17,31]. A defining feature linking obesity to T2DM is chronic, low-grade inflammation, or metaflammation, which precedes T2DM onset and drives systemic insulin resistance. This chronic inflammatory state involves the significant infiltration and activation of immune cells into key metabolic tissues like adipose tissue (AT). Several galectins are crucial modulators of this inflammation associated with metabolic disease [14,15,31,98].

Gal-3 is critical for regulating macrophage polarization within inflamed adipose tissue (AT), generally facilitating the conversion towards the reparative M2 phenotype [77]. However, the precise role of endogenous Gal-3 in metabolic inflammation remains complex and seemingly paradoxical. In high-fat diet (HFD) mouse models, targeted Lgals3 deletion exacerbated inflammation in visceral adipose tissue (VAT), evidenced by an increased presence of pro-inflammatory macrophages, and subsequently accelerated HFD-induced obesity and insulin resistance. This heightened metaflammation may be linked to Gal-3 deficiency, indirectly enhancing the activation of the NLRP3 inflammasome pathway in macrophages [91,99].

Gal-1 and -9 demonstrate immunomodulatory or anti-inflammatory activities [12,16,17]. Pharmacological inhibition of Gal-1 in HFD-fed rats resulted in reduced adipogenesis, suppressed lipogenesis, and improved metabolic variables, leading to weight loss. Furthermore, genetic deletion of Gal-1 in HFD-fed mice significantly reduced the expression of pro-inflammatory cytokines, including CCL2, CCL3, and TNFα, in both adipose and liver tissues [53]. On the other hand, Gal-9 is linked to inducing immune tolerance by prompting apoptosis in pathogenic T helper cells (Th1/Th17) and expanding regulatory T cells (Tregs) [12,16,87,94].

### 3.3. Cardiovascular and Endothelial Dysfunction

Chronic exposure to hyperglycemia leads to endothelial dysfunction via mechanisms such as oxidative stress and AGEs [27,28,59,100]. Galectins modulate key angiogenic processes by binding to and regulating receptors such as Vascular Endothelial Growth Factor Receptor 2 (VEGFR2) [26,34]. In diabetic vasculopathy, Gal-1 and Gal-3 generally exert pro-angiogenic effects [58,65]. Gal-1 is significantly upregulated in the vitreous fluid and epiretinal fibrovascular membranes of patients with proliferative diabetic retinopathy (PDR) [58]. Gal-1 demonstrates a significant positive correlation with VEGF levels in the vitreous fluid of PDR patients [26,58]. It contributes to vascular permeability through an interaction involving the neuropilin-1/VEGFR1 complex [10,22]. Elevated serum levels of Gal-3 are associated with heart failure (HF) and cardiovascular disease [29,101]. In diabetic cardiomyopathy (DCM), inhibition of Gal-3 ameliorates cardiac dysfunction, reducing myocardial apoptosis, oxidative stress, inflammation, and fibrosis. Mechanistically, Gal-3 promotes DCM by triggering the release of inflammatory cytokines and enhancing macrophage infiltration. This inflammatory signaling is regulated through the Gal-3/NF-κB p65 pathway, where Gal-3 inhibition blocks nuclear factor kappa beta (NF-κB) p65 activation [19,102,103].

The key role of galectins in various microangiopathy-related pathological conditions, including kidney, heart, and lung fibrosis has been discussed [13,33,41]. In diabetic nephropathy (DN), increased Gal-3 expression correlated with the decline in renal function and the presence of micro- and macroalbuminuria [24,104]. Gal-1 has been identified as a novel fibrosis protein that is highly expressed in renal tubular cells of kidneys from T1DM and T2DM mouse models [22,56]. The expression of Gal-3 in glomerular/mesangial tissue is markedly upregulated by the diabetic environment compared to normal conditions, often observed in infiltrating macrophages [38]. Conversely, Gal-3 serves a protective role against AGE-induced tissue injury by acting as an AGE clearance receptor, promoting the removal of these pathogenic substances. Studies using Gal-3 knockout mice showed accelerated diabetic glomerulopathy, characterized by greater AGE accumulation, suggesting impaired AGE removal in the absence of Gal-3 [105].

### 3.4. Galectins in Diabetic Neuropathy and Nerve Degeneration

Galectins are implicated in processes of neuroinflammation and neurodegeneration [11,39,67,106]. Gal-1 is expressed in the peripheral nervous system (PNS), including in Schwann cells, motor neurons, and sensory neurons [11,77,106]. Following peripheral nerve injury (axotomy), the expression of Gal-1 significantly increases in Schwann cells and dorsal root ganglia (DRG) neurons, which promotes axonal regeneration [11,12,17,107]. The oxidized form of Gal-1 (Gal-1/Ox) promotes axonal regeneration but reportedly functions distinctly from its lectin properties. In the central nervous system (CNS), Gal-1 generally contributes to nerve repair by promoting microglial polarization towards an anti-inflammatory M2 phenotype and deactivating the classically activated M1 phenotype [11,17,39].

Studies show that genetic deletion of Gal-3 in diabetic mice generally protects by preventing inner blood-retinal barrier (iBRB) dysfunction, stabilizing tight junction integrity, and reducing VEGF expression compared to wild-type diabetic controls [26,108]. Gal-3 is typically upregulated by reactive microglia/macrophages during CNS inflammation, and it promotes macrophage phagocytosis [109,110]. Conversely, Gal-1 generally functions as a neuroprotective and anti-inflammatory agent, acting to dampen inflammatory responses [17,39]. In streptozotocin-induced diabetic mice, the lack of Gal-3 attenuated neuroinflammation, protecting the retina and optic nerve and reducing retinal ganglion cell (RGC) apoptosis [108]. Schwann cells express Gal-3, and its upregulation under high glucose conditions suggests potential cytoprotective properties against diabetic injury [107]. However, pharmacological inhibition of Gal-3 using the compound combination of diosmin and hesperidin in diabetic rats significantly reduced plasma Gal-3 levels, improving motor performance and nerve regeneration, suggesting reduced Gal-3 correlates with beneficial neurological outcomes in this peripheral neuropathy model [111].

GalGal-4 is significantly involved in regulating myelination. Its expression typically decreases before the onset of myelination in the CNS, suggesting it may inhibit oligodendrocyte (OLG) differentiation. Gal-4 also modulates axonal growth and helps organize the proper localization of the neural cell adhesion molecule L1 on the axonal membrane [11,68,106]. GalGal-8 acts as a neuroprotective factor, particularly in hippocampal neurons. It defends neurons against toxic conditions associated with neurodegeneration, such as glutamate-induced excitotoxicity, oxidative stress, and Aβ aggregation [35,39].

## 4. Galectins in Diabetic Foot Ulcers

**Figure 2:**
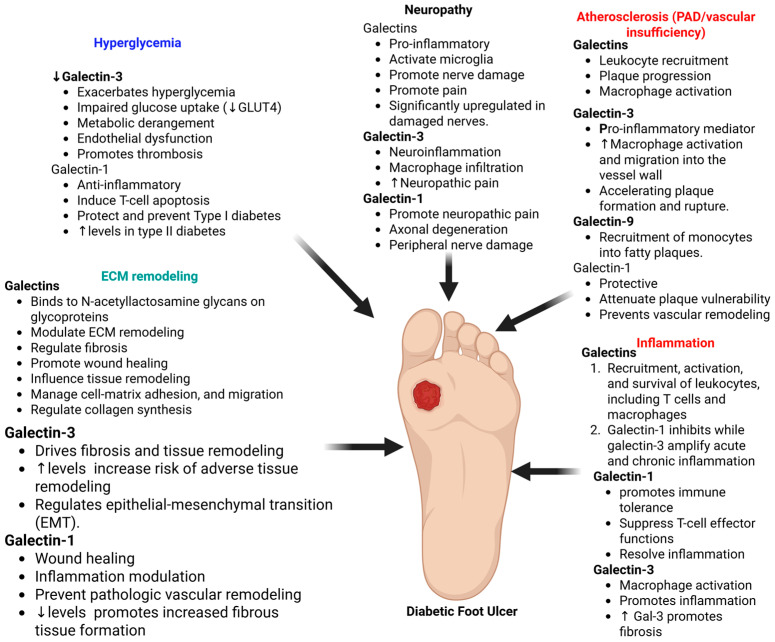
Galectins in the pathogenesis and healing of diabetic foot ulcers (DFUs). Galectins playing dual roles as amplifiers (e.g., Galectin-3) or silencers (e.g., Galectin-1) of immune response and inflammation. Galectins may contribute to hyperglycemia in type II diabetes and can protect from type I diabetes. Galectins also play a role in peripheral neuropathy and vascular insufficiency. The role of galectins in inflammation and extracellular matrix (ECM) remodeling suggests their role in DFU pathogenesis. Galectins regulatory role in these mechanisms, which are also involved in DFU pathogenesis and wound healing, is suggestive of their contribution to DFU as therapeutic targets. Since galectins, mainly galectin-3, play a critical role in DFU pathogenesis and their levels are increased, they can serve as biomarkers as well as therapeutic targets to promote wound healing. ↑ indicates increased; ↓ indicates decrease. Created in BioRender. Rai, V. (2026) https://BioRender.com/j7oxitv (accessed on 23 January 2026).

Understanding the role of galectins is critical because they bridge the gap between the systemic origin of diabetic complications and the local evolution of chronic wounds. The origin of DFUs lies in neuropathy and vasculopathy, where Gal-3 contributes to these pre-ulcerative states by amplifying atherosclerotic plaque inflammation and modulating neuroinflammatory responses that lead to sensory loss [12,109,112]. However, once ulceration occurs, the evolution toward chronicity is driven by a local failure of galectin function. Specifically, the pro-angiogenic activity of Gal-3 is competitively blocked by the accumulation of AGEs, preventing the integrin α5β1 interaction necessary for vascular repair [20,21]. Simultaneously, hyperglycemia-induced O-GlcNAcylation suppresses Gal-7, halting re-epithelialization [23,113]. Consequently, integrating these insights provides a compelling justification for targeted therapies that can circumvent molecular blockades and reinitiate wound repair in chronic ulcers [4,20]. Figure 2 depicts the role of galectins in DFU pathogenesis and the rationale of targeting them to promote healing time.

### 4.1. Normal Wound Healing: Where Galectins Act

**Figure 3:**
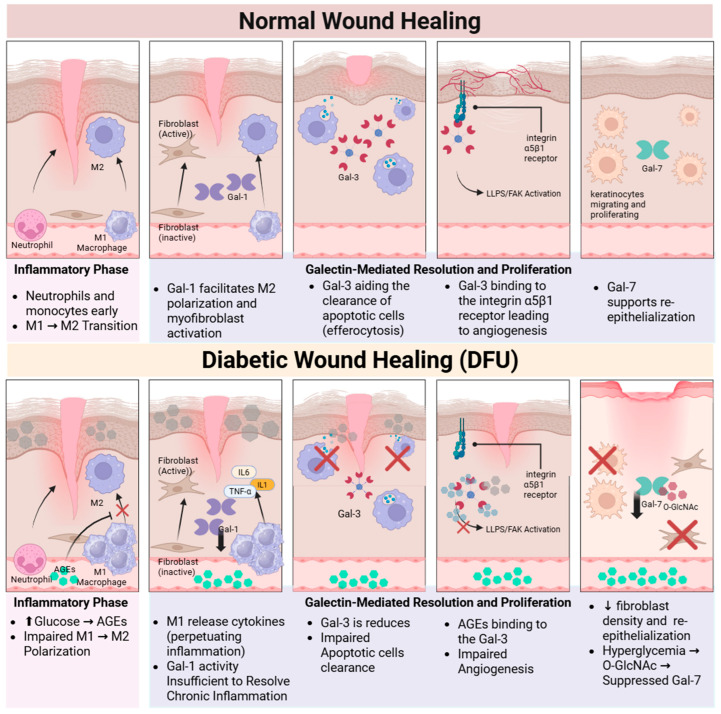
Role of galectins in normal and diabetic wound healing. Divergence between normal wound healing, characterized by timely resolution and efficient tissue regeneration, and the stalled process of a Diabetic Foot Ulcer (DFU), driven by hyperglycemia and chronic inflammation. In healthy skin, Galectin-3 (Gal-3) mediates crucial angiogenesis by activating the integrin α5β1/Focal Adhesion Kinase (FAK) pathway, while Galectin-1 (Gal-1) promotes the necessary shift from pro-inflammatory (M1) to pro-resolving (M2) macrophages. However, in the diabetic microenvironment, excessive Advanced Glycation End-products (AGEs) bind Gal-3, effectively blocking its essential regenerative interaction with integrin α5β1 and ultimately inhibiting angiogenesis and tissue formation. This molecular blockade, coupled with sustained chronic inflammation due to an impaired M1 → M2 macrophage transition, and the suppression of epithelial Galectin-7 (Gal-7), collectively perpetuates the non-healing DFU phenotype. ↑-increase and ↓-decrease. Created in BioRender. Alobaidi, A. (2026) https://BioRender.com/omchbwf (accessed on 19 December 2025).

Normal wound healing is a precise biological process encompassing four phases: hemostasis, inflammation, proliferation, and remodeling [98,114,115,116]. Galectins modulate cellular processes such as adhesion, migration, and inflammation. Gal-1 expression is upregulated during the initial phases of skin healing. During hemostasis, Gal-1 promotes platelet adhesion and aggregation by binding to the αIIbβ3-integrin receptor. As inflammation proceeds, Gal-1 induces T cell apoptosis and facilitates neutrophil migration [10,16,22,65,117,118]. Gal-3 also prompts macrophage clearance of apoptotic neutrophils (efferocytosis), which is necessary for suppressing acute inflammation and initiating the proliferative phase [16,101,119] (Figure 3).

Gal-7 primarily enhances wound re-epithelialization by regulating keratinocyte migration. Exogenous administration of Gal-7 has been shown to accelerate epithelial wound closure in mouse corneas in a manner dependent on its CRD [23,65]. Intracellular Gal-7 controls keratinocyte proliferation and differentiation through the JNK1-miR-203-p63 signaling pathway, maintaining epidermal homeostasis after injury [23,76,120]. Gal-3 boosts keratinocyte migration by binding complex N-glycans on integrin receptors, such as the α3β1 integrin, resulting in lamellipodia formation. Gal-3 also facilitates epithelial migration and rearrangement by interacting with and clustering CD147 on the cell surface, which promotes the induction of Matrix Metalloproteinase-9 (MMP-9) [65,91] (Figure 3).

The later stages of wound healing depend on specific galectins to regulate angiogenesis and ECM composition [44,65]. Gal-1 promotes neovascularization by binding to the Neuropilin-1 (NRP1) receptor on ECs, which, in turn, activates VEGFR2 signaling [22,52]. Gal-3 modulates angiogenesis induced by growth factors like VEGF and bFGF [65]. Mechanistically, this action often involves Gal-3 binding to N-glycans on the integrin αvβ3 complex, activating downstream Focal Adhesion Kinase (FAK)-mediated signaling [20,121,122]. Furthermore, Gal-1 induces the conversion of dermal fibroblasts into myofibroblasts and promotes the production of ECM components, contributing to wound contraction [22]. Similarly, Gal-3 stimulates the transformation of fibroblasts into myofibroblasts and enhances collagen synthesis, increasing wound tensile strength during repair [41,123,124] (Figure 3).

### 4.2. Specific Roles of Major Galectins in DFUs

#### 4.2.1. Galectin-1

Gal-1 is emerging as a critical factor in tissue repair processes compromised in DFUs. Gal-1 accelerates the healing of pathological wounds by promoting repair processes such as myofibroblast activation, migration, and proliferation [22,65,89,117]. This pro-healing activity is mediated through the activation of the neuropilin-1 (NRP1)/Smad3 signaling pathway, which subsequently upregulates NADPH oxidase 4 (NOX4) expression and ROS production in myofibroblasts. Studies using Gal-1 knockout (Lgals1−/−) mice demonstrated a delayed cutaneous wound healing response, confirming that the loss of endogenous Gal-1 impairs angiogenesis and the necessary repair mechanisms. Gal-1′s immune modulation capabilities are relevant for resolving the persistent chronic inflammation seen in non-healing diabetic wounds [22,52,65,89,118]. Acting as a resolution-associated molecular pattern, Gal-1 assists the transition phase by inhibiting T cell trafficking and promoting macrophage reprogramming toward the pro-resolving M2 phenotype, partly by regulating L-arginine metabolism within these immune cells [22,118]. Therefore, administering Gal-1 topically in diabetic wound models is a promising therapeutic strategy to overcome impaired DFU healing, as it reduces neutrophil infiltration and encourages the shift from pro-inflammatory M1 to pro-healing M2 macrophages [17,22].

#### 4.2.2. Galectin-3

Gal-3 circulating levels are often elevated in T2DM patients with DFU compared to both diabetic patients without ulcers and healthy controls, positioning it as a potential biomarker for DFU progression [14,18,25,125]. Gal-3 is produced by inflammatory cells, including macrophages and neutrophils, and facilitates neutrophil activation and adhesion to laminin [16,43]. In chronic diabetic wounds, Gal-3 perpetuates inflammation by functioning as a chemoattractant for monocytes and macrophages, especially at high concentrations, thus increasing their infiltration. Additionally, Gal-3 acts as an opsonin, promoting the efficient clearance of apoptotic neutrophils by macrophages, which is critical for inflammation resolution. Gal-3 facilitates the pro-inflammatory M1 phenotype in some contexts, while promoting the anti-inflammatory M2 phenotype necessary for tissue regeneration in others [119,126].

Furthermore, Gal-3 promotes fibrosis by inducing fibroblast proliferation and collagen synthesis, which can lead to tissue scarring during chronic inflammation [30,65]. Elevated Gal-3 levels in DFU patients correlate positively with inflammatory markers such as C-reactive protein (CRP) and erythrocyte sedimentation rate (ESR) [14,25,82]. Recent mechanistic studies indicate that AGEs aberrantly bind to Gal-3, disrupting its beneficial pro-angiogenic function mediated by integrin α5β1. This interference illustrates a mechanism by which Gal-3 fails to promote optimal wound healing in the high-AGE, hyperglycemic environment of chronic wounds [20,105].

#### 4.2.3. Galectin-7

Gal-7 is critical for re-epithelialization in cutaneous wound healing, a function often impaired in DFUs [65,127]. In the diabetic microenvironment, excessive hyperglycemia significantly compromises Gal-7 expression in keratinocytes. This reduction is mediated by increased intracellular O-GlcNAc glycosylation, which inhibits Gal-7 expression and reduces keratinocyte migration potential [23,91,113]. The resulting functional defect impairs wound healing kinetics because Gal-7 normally supports cell motility and maintains epidermal homeostasis after injury. Supporting its therapeutic potential, enhanced Gal-7 expression accelerates wound healing and promotes corneal epithelial closure in experimental models [23,64]. Furthermore, Gal-7 protein has been quantified within dehydrated amniotic/chorion membranes (dACM), a biological matrix utilized in treating chronic wounds [91,128]. Therefore, strategies focusing on inhibiting aberrant O-GlcNAc glycosylation or restoring Gal-7 levels present a promising novel approach for epithelial repair in diabetic patients [113].

### 4.3. Galectins, Infection, and Clinical Evidence

DFUs are significantly impacted by host susceptibility to infection and sustained inflammation [125]. Galectins function as Pattern Recognition Receptors (PRRs) by binding to microbial glycans and modulating innate immune responses and crucial signaling pathways [12,129,130]. Gal-3 binds to bacterial lipopolysaccharides (LPS) and enhances macrophage chemotaxis and phagocytosis [48,131,132]. Furthermore, Gal-3, -8, and -9 act as cytoplasmic sensors that recognize damaged phagosomes, a mechanism vital for triggering antibacterial autophagy to eliminate intracellular pathogens [12,79]. Gal-3 often displays pro-inflammatory traits, contributing to persistent, destructive inflammation by promoting the activation of components like the NLRP3 inflammasome. However, its precise effect depends on the specific context of the infection and immune cell activity [27,91,124,133].

In terms of functional demonstration, topical administration of recombinant Gal-3 embedded in hydrogels promotes enhanced wound closure and angiogenesis in diabetic rodent models [20]. This pro-angiogenic activity relies on Gal-3 binding to its functional receptor, integrin α5β1, subsequently inducing Liquid–Liquid Phase Separation (LLPS) and activating downstream Focal Adhesion Kinase (FAK) signaling required for vessel formation. Importantly, this beneficial mechanism is compromised in diabetic conditions because the aberrant accumulation of AGEs competitively binds to Gal-3, thereby preventing activation of the integrin α5β1−FAK signaling axis [20].

Serum Gal-3 levels are elevated in patients with DFUs compared to diabetic controls [14,18,25,82], yet Gal-3 expression is reduced in the skin vasculature of diabetic patients, potentially due to AGE-mediated inhibition or impaired cellular internalization [20]. In contrast, successful DFU healing is associated with increased Galectin-7 expression in keratinocytes and the presence of Gal-3-expressing healing-enriched fibroblasts (HE-Fibro) in the wound bed, a population identified by single-cell RNA sequencing and enriched in healing compared to non-healing DFUs [20,25,85]. Table 3 summarizes the role of various galectins in the pathogenesis of DFU.

## 5. Galectins: Diagnostic and Prognostic Biomarkers

Galectins function extracellularly, but they lack the canonical signal peptide required for the classical endoplasmic reticulum (ER)-Golgi secretory pathway. Instead, they are exported via non-classical secretory mechanisms, which include direct translocation across the plasma membrane, release via exosomes, and vesicular release in response to cellular stress [135,136,137]. Regarding excretion, circulating galectins are cleared by the kidneys, allowing their detection in biological fluids like serum and urine [135,138]. Gal-3 levels are significantly elevated in patients with T2DM and prediabetes compared to healthy individuals, correlating positively with HbA1c and fasting plasma glucose [14,82]. High baseline Gal-3 is an independent predictor of incident T2DM and correlates with insulin resistance indices such as HOMA-IR [54,139]. In obstetric care, circulating Gal-3 is significantly upregulated in women with GDM compared to healthy pregnant controls [100,112]. Conversely, Gal-1 levels in subcutaneous interstitial fluid increase in T2DM patients, reflecting adipose tissue inflammation and insulin resistance [95,140].

Elevated serum levels of Gal-3 found in patients with T2DM, and prediabetes positively correlate with indicators of poor glycemic control, such as HbA1c [14,18,82]. In diabetic kidney disease (DKD), high circulating Gal-3 levels correlate inversely with estimated Glomerular Filtration Rate (eGFR), predicting severe disease progression [24,141,142]. High Gal-3 levels are also linked to adverse outcomes in DKD, such as the doubling of serum creatinine and macroalbuminuria [18,24]. Similarly, high serum Gal-1 is an independent predictor of renal function decline in patients undergoing coronary angiography [57]. Urinary Gal-3 also serves as a specific, non-invasive biomarker correlating with renal interstitial fibrosis and tubular injury severity [24,143]. A study with 556 patients investigated the association between plasma levels of Gal-3 and uric acid (UA) with a decline in renal function and the results suggested that UA and Gal-3 plasma levels are independent positive predictors of a decrease in eGFR in patients with CAD and normal or mildly reduced renal function [144]. Another study with 964 patients investigated for the biomarkers predicting cardiovascular events in patients with T2DM and atherosclerosis. The study reported that increased Gal-3 levels are associated with cardiovascular events in patients with CAD and T2DM [145]. In terms of the elevated levels of serum Gal-3 and renal dysfunction, Karolko et al. reported that circulating Gal-3 has no prognostic usefulness in heart failure patients with preserved ejection fraction associated with moderate renal dysfunction. However, increased Gal-3 levels are associated with exercise intolerance with impaired renal function [146]. These studies suggest the importance of Gal-3 as prognostic indicator. While Gal-3 diagnostic value is recognized, there is a need for ongoing research into its role as a causative mediator of fibrosis and inflammation, necessitating preclinical and clinical studies that block its function to verify its therapeutic potential.

For DFUs, serum Gal-3 levels are significantly higher in patients with active ulcers compared to diabetic controls [14,25]. This systemic elevation contrasts with reduced local Gal-3 expression in the diabetic skin vasculature, highlighting a spatial discrepancy in DFU pathology [20]. Furthermore, Gal-3 is a potent predictor of all-cause and cardiovascular mortality in patients with T2DM [97]. Additionally, elevated serum Gal-4 is associated with heart failure and prevalent diabetes, potentially reflecting intestinal barrier dysfunction [68,83].

## 6. Therapeutic Strategies Targeting Galectins

Therapeutic interventions involving galectins require a dual approach: systemic inhibition to mitigate metabolic dysfunction and fibrosis, and local supplementation to accelerate wound healing in DFUs [4,13]. Therapeutic strategies targeting galectins typically use small-molecule carbohydrate inhibitors (glycomimetics) or large natural polysaccharide derivatives [13,30,33,147]. To counteract the pro-inflammatory and fibrotic actions of circulating galectins, pharmacological development has focused on high-affinity inhibitors that block CRD [13,30]. TD139 (GB0139), a potent thiodigalactoside inhibitor originally designed for pulmonary fibrosis [148], has demonstrated significant efficacy in reducing hyperglycemia and inflammation in diabetic mouse models [149]. In murine models of GDM, TD139 administration successfully alleviated hyperglycemia and suppressed placental inflammation by inhibiting the ERK/JNK/p38 signaling pathways [149]. Building on this scaffold, GB1211 was developed as the first orally bioavailable Gal-3 inhibitor, which is currently under clinical evaluation for liver cirrhosis and metabolic dysfunction-associated steatotic liver disease (MASLD) [33].

Additionally, Modified Citrus Pectin (MCP), capable of binding Gal-3, has been shown to ameliorate diabetes-associated cognitive impairment and neuroinflammation in vivo by reducing oxidative stress [150]. Another polysaccharide antagonist, Belapectin (GR-MD-02), has been evaluated in clinical trials for non-alcoholic steatohepatitis (NASH), showing benefits in specific patient subgroups with portal hypertension [151]. Furthermore, targeting Gal-1 with the specific inhibitor OTX008 has proven effective in pre-clinical models of proliferative diabetic retinopathy, where it attenuates VEGF-induced endothelial migration and pathological neovascularization [58].

In the specific microenvironment of DFUs, the therapeutic objective shifts from inhibition to the restoration of functional galectin levels. Although systemic Gal-3 is elevated, its local pro-angiogenic activity within the wound bed is competitively blocked by the accumulation of AGEs [20]. To override this blockade, recombinant Gal-3 encapsulated in hydrogels has been developed for topical application. This localized delivery system restores the critical interaction between Gal-3 and the integrin α5β1 receptor, triggering liquid–liquid phase separation (LLPS) and activating Focal Adhesion Kinase (FAK) to drive robust angiogenesis. Notably, the co-administration of Gal-3 with insulin in these hydrogels yields a synergistic acceleration of wound closure and collagen deposition without inducing systemic insulin resistance [20]. Similarly, the topical application of Gal-1 accelerates wound healing by facilitating the transition of macrophages from the pro-inflammatory M1 phenotype to the regenerative M2 phenotype and enhancing myofibroblast activation via the Neuropilin-1/Smad3 pathway [22,28,76]. Inhibiting Gal-1 with the small molecule OTX008 attenuated VEGF-induced endothelial cell migration and restricted retinal neovascularization in rat models of DR. This places Gal-1 inhibition as a complementary pharmacological tool alongside anti-VEGF agents used in proliferative diabetic retinopathy (PDR) therapy [13,26,58]. Conversely, targeting Galectin-3 (Gal-3) aims to reverse insulin resistance and inflammation in insulin-sensitive organs affected by T2DM [14,15,30,92]. Acute administration of recombinant Gal-1 exerted protective, anti-inflammatory effects following renal ischemia–reperfusion injury, suggesting recovery potential in diabetes-related vascular complications [11,16,22,54]. Collectively, these approaches demonstrate that clinical efficacy depends on route-specific modulation of galectin activity, balancing systemic antagonism to prevent metabolic dysfunction with localized restoration to drive wound repair. Table 4 summarizes the clinical and therapeutic relevance of various galectins in diabetic foot ulcers.

## 7. Challenges and Knowledge Gaps

Current biomarker and methodological studies concerning galectins in diabetes and DFU face fundamental limitations stemming from assay inconsistency and the inherent nature of clinical cohorts [24,26]. The clinical utility of galectin biomarkers is constrained because few human studies employ the extensive longitudinal designs necessary to monitor dynamic activity across disease stages [83]. This limits the capacity to infer causal relationships between galectin levels and disease progression. Furthermore, technical constraints hinder biomarker evaluation; measuring circulating galectins is complicated by heterogeneous methodologies, contrasting results obtained from different sample types (serum versus plasma), and the absence of standardized protocols [58,112,152]. Even in automated classification systems for DFU, performance is mediocre due to small, imbalanced, and limited publicly available image datasets, restricting the reliability and generalizability of prediction models [153].

The relationship between Gal-3 and AGEs remains mechanistically controversial, as studies show Gal-3 acts as a protective scavenger receptor for AGEs in the kidney but is implicated in AGE-disrupted pro-angiogenic signaling that impairs wound healing [20]. Moreover, the temporal dynamics of galectin signaling during wound healing phases require further clarification, as illustrated by the distinct roles of certain galectins during early versus late stages of retinal ischemic injury [26]. Furthermore, developing selective inhibitors is challenging because the CRD is conserved across different galectin family members, increasing the risk of unintended off-target effects [45,154]. Many promising carbohydrate-based ligands or glycomimetics exhibit poor pharmacological properties due to low metabolic stability and high hydrophilicity [26,45]. Regulatory obstacles, particularly in the case of complex polysaccharide inhibitors such as GCS-100, involve stringent requirements from agencies like the Food and Drug Administration to demonstrate the discrete contribution of each component before advancing to late-stage trials [30,155].

Methodological gaps persist regarding the limitations of current preclinical models in replicating complex human diabetic pathology. Rodent DFU models fail to reproduce critical features observed in human diabetic foot ulcers, including chronic neuropathic progression and the complex biomechanical pressures experienced on the plantar surface [156]. To overcome these limitations, future research demands a shift towards employing single-cell and spatial profiling, which are necessary to map the intricate molecular composition and cellular activities within the wound microenvironment [26,157]. Broadening the scope of clinical evidence requires systematic integration of biomarker data and multi-center trials incorporating diverse populations [24,158]. The pathway to integrating galectin findings into personalized diabetes care requires developing strategies for patient stratification and precision targeting to optimize therapeutic benefits [88,157].

Personalized medicine approaches could involve combining circulating galectin levels with clinical risk factors (e.g., age or education) to create validated nomograms, enhancing the ability to predict microvascular complications such as mild cognitive impairment in T2DM patients [159]. Given the challenges of systemic administration, adopting precision delivery methods, such as local therapeutic application via hydrogels, offers a viable strategy to achieve high local concentrations of galectin antagonists or recombinant proteins while minimizing systemic exposure and side effects [4,20,22]. Finally, the high correlation between galectins and components of the immune checkpoint cascade suggests that co-targeting galectins (e.g., Gal-1 inhibition) in combination with existing immunotherapies presents a promising strategy for enhancing anti-tumor effects [154,160]. The central challenge in galectin-targeted therapy is its context-dependent duality. This pleiotropy limits systemic inhibition, making selective, tissue-specific targeting essential to suppress disease-driving activity while preserving physiological function.

## 8. Future Directions

The complexity surrounding the role of galectins in diabetes and DFUs necessitates therapeutic strategies that prioritize specificity and precise delivery [6,13,14,105]. Developing selective inhibitors remains challenging due to the conserved nature of the CRD shared across the galectin family [13,33,45,154]. To mitigate the risks associated with systemic exposure, particularly related to Gal-3-induced insulin resistance, future research must emphasize local therapeutic application through advanced delivery systems like hydrogels [4,14,20,161]. This ensures that therapeutic concentrations are maximized directly at the ulcer site to promote angiogenesis and collagen deposition [22].

Achieving improved diagnostics and effective patient stratification requires a shift toward molecular understanding [13,26]. Researchers should utilize scRNA-seq and spatial profiling to map the heterogeneous cellular landscape of DFU tissue. This approach is essential for identifying specific dysfunctional immune cell phenotypes, such as altered macrophage polarization, and characterizing pro-healing fibroblast subpopulations unique to healing outcomes [85,162]. This molecular profiling must be integrated into longitudinal cohort studies to validate galectins as robust biomarkers for early disease detection and prognosis [24,83].

Finally, therapeutic strategies must overcome the limitations of preclinical models, which fail to replicate the complex neuropathic and biomechanical elements characteristic of human DFUs. The development of sophisticated bioengineered tissue constructs or organ-on-a-chip systems is necessary for rigorous preclinical validation of novel galectin-targeted therapies. Ultimately, future success lies in combinatorial approaches, integrating galectin modulation strategies with controlled-release growth factors, stem cell therapies, and targeted anti-biofilm agents [6,27,125,156,161,163].

## 9. Conclusions

Galectins, particularly Gal-3, are pivotal yet contextually complex regulators linking systemic metabolic dysfunction to localized impaired tissue repair observed in DFUs. Systemically, elevated serum Gal-3 serves as a critical biomarker correlating positively with insulin resistance, chronic inflammation, and the prognosis of complications like diabetic nephropathy and cardiomyopathy. However, a profound local paradox compromises repair: Gal-3′s essential pro-angiogenic function, driven by binding to the integrin α5β1 receptor and the resultant FAK signaling following LLPS, is blocked by the excessive accumulation of AGEs within the diabetic microenvironment. This wound chronicity is compounded by the suppression of Gal-7 expression in keratinocytes via hyperglycemia-induced O-GlcNAc glycosylation, thereby impeding necessary re-epithelialization. Therefore, successfully addressing DFU pathogenesis requires moving toward spatially precise therapeutic modulation, such as the local application of recombinant Gal-3 encapsulated in hydrogels to promote vessel formation and subsequent healing, coupled with advanced molecular profiling like scRNA-seq to ensure patient stratification and targeted regenerative interventions.

## Figures and Tables

**Table 1:** Galectins and their role in diabetes and diabetic foot ulcers (DFUs). Carbohydrate recognition domain (CRD), vascular endothelial growth factor (VEGF), type 2 diabetes mellitus (T2DM), advanced glycation end products (AGEs).

Galectin	Major Tissue Expression	Key Biological Functions	Relevance to Diabetes/DFUs
Gal-1 (Prototype (single CRD, forms homodimer) [22,53,54,58]	Expressed in Epithelium, Vascular/Endothelial cells, Adipose tissue, Immune cells	Immunosuppression (induces activated T cell apoptosis, promotes M2 macrophage polarization); Pro-angiogenic factor Promotes myofibroblast activation; Regulates cell proliferation and migration	Circulating levels are often increased in T2DM patients and correlate with insulin resistance. Accelerates pathological wound healing in diabetic models by promoting myofibroblast activation via NRP1/Smad3 signaling. Upregulated in diabetic retinopathy (PDR) as a pro-angiogenic factor correlating with VEGF. Supports pancreatic insulin secretion,
Gal-2 Prototype (single CRD, forms homodimer) [60,62]	Predominantly in the gastrointestinal epithelium, also found in cardiovascular tissue and the placenta	Supports mucosal barrier integrity (mucin crosslinking); Induces T-cell apoptosis; Can drive pro-inflammatory M1 macrophage polarization (context dependent)	Genetic variants linked to elevated fasting glucose and insulin levels. Placental expressions are often regulated in Gestational Diabetes Mellitus (GDM)
Gal-3 Chimera (single CRD + proline/glycine-rich N-terminal domain, forms oligomer/pentamer) [20,30,82]	Expressed in Epithelial cells, Macrophages/Monocytes/Neutrophils, Fibroblasts, Heart, Kidney, and Liver	Strong Pro-inflammatory and Pro-fibrotic mediator (activates macrophages/fibroblasts); Angiogenesis; Anti-apoptotic (intracellular); Acts as an innate immune pattern recognition receptor (PRR); Binds AGEs (scavenger)	Systemically elevated in obesity and T2DM. Correlates with insulin resistance by directly binding to the insulin receptor. Locally in DFUs, its crucial pro-angiogenic function is disrupted by accumulating AGEs. Elevated serum levels are prognostic biomarkers for complications.
Gal-4 Tandem repeat (two distinct CRDs, tandem) [68,69,83,84]	Highly concentrated in Gastrointestinal Epithelium	Stabilizes lipid rafts; Regulates apical protein trafficking (DPP-4); Modulates intestinal inflammation; Promotes intestinal wound healing	Elevated serum linked to diabetes/CVD/ischemic stroke risk. May influence glucose homeostasis by enhancing DPP-4 activity, potentially reducing GLP-1 (Glucagon-Like Peptide-1) action. Associated with obesity primarily in diabetic patients.
Gal-7 Prototype (single CRD, forms homodimer/monomer) [23,85]	Highly concentrated in Stratified epithelia (Skin Keratinocytes, Cornea)	Essential for Epithelial homeostasis; Promotes re-epithelialization by enhancing keratinocyte migration and proliferation; Regulates apoptosis.	Expression is suppressed by hyperglycemia in diabetic keratinocytes, leading to impaired epithelial repair. High expression associated with successful DFU healing (DFU-Healers). Exogenous application stimulates wound closure.
Gal-8 Tandem-repeat (two distinct CRDs, tandem) [35,39]	Expressed in the Liver, Kidney, Brain, Adipose tissue, and Immune cells	Endomembrane damage sensor (targets damaged vesicles for autophagy); Promotes cell adhesion/migration; Neuroprotective factor; Induces T-cell apoptosis	Identified as a neuroprotective factor in the CNS, defending hippocampal neurons against excitotoxicity and oxidative stress. Senses endomembrane damage, a mechanism critical for antibacterial defense.
Gal-9 Tandem-repeat (two distinct CRDs, tandem) [72,86,87]	Immune tissues (Thymus, Liver, Spleen), Small intestine, Kidney	Induces immune tolerance (promotes apoptosis of Th1/Th17 cells via TIM-3); Senses membrane damage/promotes autophagy; Regulates glucose metabolism	Plays a role in glucose homeostasis by mediating GLUT-2 transporter retention on β-cell surfaces. Elevated serum levels are seen in T2D/CKD patients. Dysregulated concentration observed in Gestational Diabetes Mellitus (GDM).
Gal-10 Prototype (single CRD) [61]	Eosinophils, Basophils, Placenta	Associated with eosinophil/basophil inflammation; Functions in T-regulatory cells; Associated with Charcot-Leyden crystals	Overexpressed in the nuclear decidua and syncytiotrophoblast of GDM placentas.
Gal-12 Tandem repeat (two distinct CRDs, tandem) [61]	Predominantly expressed in Adipose tissue	Negative regulator of lipolysis (acting on lipid droplets); Induces adipocyte apoptosis; Promotes pro-inflammatory M1 macrophage polarization	Ablation improves insulin sensitivity and reduces adiposity/glucose tolerance in obese animal models. Enhances inflammation by promoting M1 macrophage polarization. Elevated in GDM placental tissue.

**Table 2:** Roles of Galectins in Metabolic Dysregulation. T2DM- type 2 diabetes mellitus, GDM- gestational diabetes mellitus, PPARγ- peroxisome proliferator-activated receptor gamma, LPS- lipopolysaccharides, glucose transporter type 1 (GLUT1), hexokinase 2 (HK2), and pyruvate kinase M2 (PKM2).

Galectin	Target Tissue	Metabolic Effect	Mechanism
Galectin-1 [22,34,53,54,55]	Adipose Tissue, Pancreatic β-cells, Renal Tubular Cells	Accelerates high-fat diet (HFD)- induced obesity and lipogenesis; negatively associated with fasting glucose; enhances glucose-stimulated insulin secretion (GSIS) from pancreatic β-cells; elevated levels are associated with lower renal function.	Promotes adipogenesis by interacting with and activating PPARγ transcriptional activity (adipose tissue); deficiency results in reduced expression of lipogenic genes (adipose tissue); enhances β-cell insulin release (paracrine effect); promotes kidney fibrosis via the Akt/AP4 signaling pathway under hyperglycemia.
Galectin-2 [60,62]	Placenta, Cardiovascular Tissue	Placental expression is often upregulated in Gestational Diabetes Mellitus (GDM); genetic variants are linked to elevated fasting glucose and insulin levels.	Genetic polymorphisms (SNP rs7291467) in the encoding gene (LGALS2) correlate with higher fasting levels of glucose and insulin; they may influence M1 macrophage polarization.
Galectin-3 [14,43,82,90,91,92,93]	Macrophages, Adipocytes, Hepatocytes, Myocytes, Pancreatic β-cells	Causes systemic Insulin Resistance and glucose intolerance; elevated in obesity, T2DM, prediabetes, and GDM; contributes to β-cell apoptosis (when overexpressed in β-cells); may promote glycolysis.	Extracellular Gal-3 directly binds the Insulin Receptor (InsR), inhibiting tyrosine phosphorylation and downstream signaling (IRS1/AKT); intracellular Gal-3 acts as an LPS sensor to activate the mTORC1 pathway, promoting glycolysis (upregulating GLUT1, HK2, PKM2); secreted Gal-3 impairs β-cell function by inhibiting calcium channels (CACNG1).
Galectin-4 [68,69,83,84]	Gastrointestinal Epithelium, Placenta	Associated with an increased likelihood of diabetes and obesity (primarily in diabetic patients); positively correlated with Fasting Plasma Glucose (FPG) and the incretin GIP.	Regulates the apical trafficking of Dipeptidyl Peptidase-4 (DPP-4) in enterocytes, potentially enhancing DPP-4 activity and inactivating GLP-1/GIP hormones; binds to CD14 on monocytes, promoting differentiation into macrophages.
Galectin-9 [16,72,86,87,94]	Pancreatic β-cells, Adipose tissue, Placenta	Regulates glucose homeostasis in T2DM (via transporter function); elevated levels are seen in T2DM patients; ablation exacerbates maternal glucose intolerance in GDM mouse models.	Favors retention of the GLUT-2 glucose transporter on the pancreatic β-cell surface to sustain GSIS; plays a role in regulating hepatic NKT cell homeostasis via the Tim-3 pathway; ablating it impairs autophagy in placental cells.
Galectin-12 [59,73,74,75]	Adipocytes, Macrophages	Deficiency increases lipolysis and improves insulin sensitivity and glucose tolerance in obese animal models; promotes M1 macrophage polarization.	Functions as an intrinsic negative regulator of lipolysis (localized on lipid droplets), promoting lipid accumulation; indirectly reduces insulin sensitivity in adipocytes by fostering M1 (pro-inflammatory) macrophage polarization.

**Table 3:** Galectins in Inflammation and Wound Healing. NADPH oxidase 4 (NOX4), neuropilin-1 (NRP1), lipopolysaccharide (LPS), advanced glycation end product (AGE), epidermal growth factor receptor (EGFR), transforming growth factor (TGF)-β, diabetic foot ulcer (DFU), interleukin 6 (IL-6), C-X-C motif chemokine ligand 1 (CXCL1), T cell immunoglobulin and mucin domain-containing protein 3 (TIM-3).

Galectin	Cell Type Affected	Effect on Inflammation	Impact on Wound Healing	Evidence Type (Animal/Human/In Vitro)
Galectin-1 (Gal-1) [17,22,44,58,59,117]	T cells (activated, Th1/Th17), Macrophages (M1/M2), Myofibroblasts, Endothelial cells, Neutrophils, Platelets	Anti-inflammatory/Immunosuppressive; Induces T cell apoptosis; Promotes macrophage shift toward pro-resolving M2 phenotype; Inhibits neutrophil recruitment.	Accelerates pathological wound healing (diabetic models); Promotes myofibroblast activation, migration, and proliferation (via NRP1/Smad3/NOX4); Essential for angiogenesis/neovascularization; Deficiency delays healing; Promotes platelet adhesion/aggregation	Human (PDR, vitreous, plasma, skin), Animal (Diabetic/Excisional/Ischemia models in rats/mice), In vitro (T cells, Macrophages, Endothelial cells, Fibroblasts)
Galectin-2 (Gal-2) [31,60,62,65,120,134]	T lymphocytes, Monocytes/Macrophages (M1), Gastrointestinal epithelial cells	Induces T cell apoptosis (via caspase-3/9); Can induce pro-inflammatory M1 macrophage phenotype (context-dependent); Regulates inflammation by binding lymphotoxin-α	Promotes epithelial wound healing/mucosal healing in the gastrointestinal tract; Associated with altered glucose/insulin regulation (GDM)	Human (Placenta, Cardiovascular tissue), Animal (Mouse colitis model), In vitro (T cells, Monocytes, Epithelial cells)
Galectin-3 (Gal-3) [20,25,27,123,132]	Macrophages (M1/M2), Monocytes, Neutrophils, Fibroblasts, Keratinocytes, Endothelial cells	Pro-inflammatory mediator (activates inflammatory cells, binds LPS); Functions as an opsonin (enhances clearance of apoptotic neutrophils); Drives fibrosis/scarring; Elevated circulating levels correlate with chronic inflammation	Promotes angiogenesis (via Integrin α5β1 signaling); Promotes re-epithelialization (keratinocyte migration via EGFR/ALIX); Promotes collagen synthesis/wound tensile strength; Impaired function in diabetic wounds due to AGE binding	Human (Serum, DFU lesions, atherosclerotic plaque), Animal (Diabetic/Excisional models, MI models), In vitro (Neutrophils, Macrophages, Keratinocytes, Endothelial cells)
Galectin-4 (Gal-4) [60,65,69,83,120]	Gastrointestinal epithelial cells, Monocytes	Promotes monocyte differentiation toward macrophage-like cells	Promotes intestinal epithelial wound healing/closure (TGF-β-independent)	Human (Serum), In vitro (Epithelial cells, Monocytes)
Galectin-7 (Gal-7) [23,44,64,113]	Keratinocytes, T cells, Periodontal Ligament Fibroblasts	Modulates keratinocyte apoptosis and proliferation in response to injury; Induces T cell apoptosis	Crucial regulator for re-epithelialization; Promotes keratinocyte migration; Expression is significantly decreased in diabetic keratinocytes (impaired DFU healing); Exogenous application stimulates epithelial wound closure	Human (Skin, Scars), Animal (Mouse/Rat skin/corneal injury models), In vitro (Keratinocytes)
Galectin-8 (Gal-8) [35,65,71,79]	Microvascular Endothelial cells, Neutrophils, T cells	Cytoplasmic sensor activating antibacterial autophagy; Promotes extracellular pro-inflammatory cytokine/chemokine secretion (e.g., IL-6, CXCL1); Induces T-cell apoptosis	Pro-angiogenic factor (promotes endothelial cell migration/sprouting); Mediates fibrogenesis	Animal (Mouse model), In vitro (Endothelial cells, T cells)
Galectin-9 (Gal-9) [12,16,65,72,79,86]	T cells, Th1/Th17/Treg Macrophages (M1/M2)	Immunosuppressive/Anti-inflammatory; Induces immune tolerance/apoptosis in T cells (via TIM-3); Cytoplasmic sensor for antibacterial autophagy; Can suppress M1 macrophage polarization	Promotes angiogenesis; Plays a role in epithelial restitution	Animal (Mouse models), In vitro (T cells, Macrophages)

**Table 4:** Clinical and Translational Relevance of galectins in diabetic foot ulcers. Type 2 diabetes mellitus (T2DM), diabetic foot ulcers (DFU), myocardial infarction (MI), heart failure (HF), ischemia–reperfusion (IR), Nonalcoholic Steatohepatitis (NASH).

Galectin	Sample Type	Clinical Association	Potential Use (Biomarker/Therapy)	Limitations
Galectin-1 (Gal-1) [13,17,22,54,56,57,58]	Serum, Plasma, Tissue (Renal tubular cells, Endothelial cells, Vitreous fluid)	Elevated levels associated with insulin resistance and progression of kidney function decline (CKD) are significantly elevated in the vitreous fluid of Proliferative Diabetic Retinopathy (PDR) patients.	Targeted therapy (e.g., OTX008) for PDR by inhibiting pro-angiogenic activity. Potential therapeutic targeting for renal fibrosis in diabetes. Recombinant protein accelerates pathological wound healing and promotes pro-resolving M2 macrophage polarization.	Pleiotropy and context-dependent duality complicate systemic administration. High homology of the binding domain (CRD) challenges specific inhibitor design.
Galectin-3 (Gal-3) [13,20,25,29,30,151]	Serum, Plasma, Tissue (DFU lesions, Kidney, Placenta)	Systemically elevated in T2DM, prediabetes, Gestational Diabetes Mellitus (GDM), DKD, and DFUs. Elevated serum is strongly correlated with disease progression in DKD and heart failure (HF).	Critical Biomarker for the prognosis of DKD, HF, and DFU progression. Therapeutic strategies target systemic metabolic disorders and fibrosis (e.g., NASH) via inhibitors like Belapectin (GR-MD-02) and TD139 (GB0139). Localized recombinant Gal-3 therapy (in hydrogels) promotes angiogenesis in DFUs.	Systemic inhibition is limited by its dual role (IR inducer vs. local regenerative factor). The DFU repair function is blocked locally by Advanced Glycation End-products (AGEs). Inhibitor trials have shown mixed clinical efficacy for some indications.
Galectin-7 (Gal-7) [23,64,85,113]	Tissue (Keratinocytes, DFU lesions, Placenta), Serum	Tissue expression is suppressed in diabetic keratinocytes due to O-GlcNAc modification, compromising re-epithelialization. High expression in DFU tissue often correlates with successful healing.	Therapeutic target for accelerating re-epithelialization; strategies focus on restoring its local function. Exogenous application promotes epithelial wound closure.	Susceptible to down-regulation by hyperglycemia. Local deficiency is a key component of impaired wound healing in DFUs.
Galectin-9 (Gal-9) [13,16,72,86,87,94]	Serum, Plasma	Elevated plasma levels associated with T2DM and CKD progression. Elevated levels are linked to the severity of acute/chronic infectious diseases. Dysregulated expression noted in GDM.	Potential Biomarker for disease activity and severity, including infectious diseases. Plays a role in glucose homeostasis by supporting GLUT-2 retention on β-cell surfaces. Potential Therapeutic target for immune modulation/tolerance.	Challenges exist in developing highly selective inhibitors due to conserved CRD structure.

## Data Availability

No new data were created or analyzed in this study.
